# Retraction: HDAC regulates the diabetic vascular endothelial dysfunction by targeting MnSOD

**DOI:** 10.1042/BSR20181042_RET

**Published:** 2026-04-02

**Authors:** Zehao Liu, Qian Hou, Ke Hu, Xiaofeng Liu, Jiao Quan

**Affiliations:** Department of Endocrinology, Xiangya Hospital, Central South University, 87 Xiangya Road, Changsha 410008, Hunan, China

**Keywords:** apoptosis, cell proliferation, diabetes, endothelial cells, HDAC2, MnSOD

This article is being retracted from *Bioscience Reports* at the request of the authors following receipt of a notification from a reader alerting the Editorial Office to similarities that exist within the manuscript and between this article and another published paper with no common authors.

Specifically, duplications were noted between the GAPDH bands in Figure 2A and Figure 3G of the article. Additionally, the BAX and Cleaved caspase 3 bands in Figure 3G appear similar to the Figure 3B TRIM59 band and Figure 2F TRIM59 and SOX9 bands from Sang et al. (2018) (DOI: 10.1158/0008-5472.CAN-17-2774).

The authors are unable to provide the original raw data and therefore wish to retract the article. The Editor-in-Chief and Editorial board agree with the retraction.

